# The Partial Duplication of the 5′ Segment of *KMT2A* Revealed *KMT2A*-*MLLT10* Rearrangement in a Boy with Acute Myeloid Leukemia

**DOI:** 10.1155/2017/6257494

**Published:** 2017-12-28

**Authors:** Hiroko Fukushima, Toru Nanmoku, Sho Hosaka, Yuni Yamaki, Nobutaka Kiyokawa, Takashi Fukushima, Ryo Sumazaki

**Affiliations:** ^1^Department of Child Health, Faculty of Medicine, University of Tsukuba, Ibaraki, Japan; ^2^Department of Clinical Laboratory, University of Tsukuba Hospital, Ibaraki, Japan; ^3^Department of Pediatrics, University of Tsukuba Hospital, Ibaraki, Japan; ^4^Department of Pediatric Haematology and Oncology Research, National Research Institute for Child Health and Development, Tokyo, Japan

## Abstract

The duplication of 5′ segment of *KMT2A* is a rare molecular event in childhood leukemia, and the influence on prognosis is unknown. Here, we report on a boy who developed acute monocytic leukemia. Fluorescence in situ hybridization revealed the duplication of the 5′ segment with 2 normal alleles at *KMT2A* which was eventually found to be fused with *MLLT10*. Chemotherapy promptly induced the first complete remission in the patient at our facility, and the patient remained in first complete remission with negative minimal residual disease at 3.5 years from diagnosis. Our case is similar to two previously reported patients who had partial duplication of the 5′ segment of *KMT2A* with a *KMT2A*-*MLLT10* rearrangement. Further studies and experience with this cryptic translocation may shed more light on the management of acute myeloid leukemia.

## 1. Introduction

The histone-lysine *N*-methyltransferase 2A enzyme (MLL1), encoded by the *KMT2A* gene, is an upregulator of global, hematopoietic gene transcription, and translocation rearrangement within *KMT2A* causes variable risk stratification in acute leukemia based on the final genetic outcome.

Patients with *MLL* rearrangement other than t(9;11) and t(11;19) have an inferior outcome [1], and there are additional aberrations in *KMT2A* rearrangement, such as fusion with preferential partner gene *MLLT10*, that also carry prognostic significance [2].

One of these aberrations is the duplication/amplification of the 5′ segment of *KMT2A,* which is a very rare molecular event, and the influence of this on patient prognosis is unknown. However, in general, *KMT2A* amplification as an acquired genetic aberration has been reported to result in a poor prognosis [3]. With this deleterious effect of *KMT2A* overexpression in acute leukemia in mind, it is possible that even partial amplification could affect the patient's prognosis. In the literature, only 2 cases have been reported: one pediatric case of AML-M5b(FAB: French-American-British Classification) with the duplicated 5′ segment of *KMT2A* relapsed 16 months after diagnosis during maintenance therapy and was later salvaged by allogeneic transplantation [4] and an adult case of AML-M5a(FAB) with an amplified 5′ part of 11q23.3 where *KMT2A* was located eventually needed transplantation [5]. In both literature cases, this partial duplication was paired with *KMT2A*-*MLLT10* rearrangement.

Although preferential fusion with *MLLT10* has been well documented, the isolated prognostic importance of the partial amplification of *KMT2A* remains unknown. Here, we present the case of a pediatric male patient with AML who was successfully treated by multiagent chemotherapy alone. The 5′ duplication of *KMT2A* was identified by fluorescence in situ hybridization (FISH) before treatment, but fusion to *MLLT10* was discovered by RNA sequencing after completion of the treatment even though reverse transcription-PCR at the diagnosis did not detect any fusion partners.

## 2. Case Description

**Figure 1:**
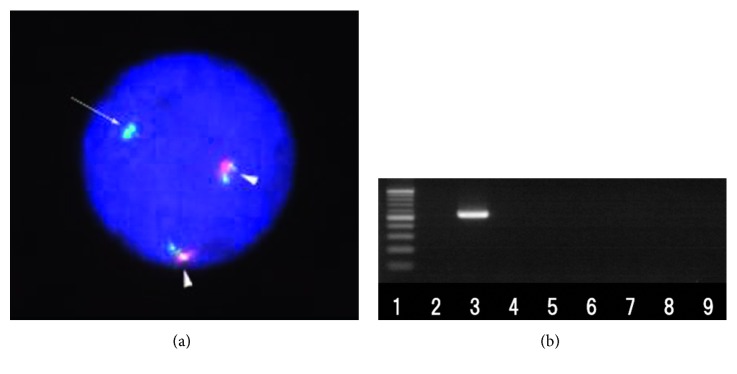
FISH and RT-PCR of the *KMT2A* rearrangement. (a) FISH of the *KMT2A* break apart signal: the green signal shows the 5′ segment, and the red signal shows the 3′ segment of *KMT2A*. FISH revealed 2 normal signals (arrowhead) and 1 additional 5′ segment (arrow). Positive cells with duplication of the 5′ segment of *KMT2* were observed in 686 out of 1000 cells. (b) The characterization of *KMT2A*-*MLLT10* fusion transcript at bone marrow by RT-PCR. 1: ladder; 2: normal control; 3: at diagnosis; 4: after induction therapy 1; 5: after induction therapy 2; 6: after intensification 1; 7: after intensification 2; 8 and 9: after intensification 3.

A 6-year-old boy was admitted with complaints of low-grade fever, multiple joint pain, skin rash, and neutropenia. A complete blood count was conducted; leukocytes were 1.6 × 10^9^/L, hemoglobin was 9.1 g/dl, and platelet count was 222 × 10^9^/L. The bone marrow was replaced by 90% monoblasts. Flow cytometry was conducted on the leukemic cells. HLA-DR, CD58, CD99, CD56, CD38, cy-MPO, CD11b, CD13, CD33, CD65, CD64, CD117, CD36, CD61, CD4, and 7.1 were positive, and CD14, CD15, CD19, CD10, CD20, CD3, and CD7 were negative. He was diagnosed with AML (FAB M5a). FISH analysis using a *KMT2A* locus-specific dual-color DNA probe (Vysis LSI MLL Dual Color, Break Apart Rearrangement Probe, Abbott Laboratories, IL, USA) was used to characterize the partial 5′ duplication and 2 other normal *KMT2A* alleles (Figure 1). Cell culture for chromosomal analysis failed. The search for *KMT2A*-*MLLT10* fusion was performed according to a publication previously reported [6]. Reverse transcription-polymerase chain reaction of major fusion partners to *KMT2A* (including *MLLT10*) was conducted and none were amplified. The primer for *KMT2A* was designed in exon 8.

Final diagnosis, in this case, was AML with 11q23/*KMT2A* abnormalities (FAB M5a) without any confirmation of partner genes. As he presented no abnormalities such as t(8;21), inv(16), -7, 5q-, t(16;21) (p11;q22), Ph1, and *FLT3*-*ITD*, he underwent multimodal chemotherapy for the intermediate risk group according to JPLSG AML05, which consists of induction 1 (ECM: etoposide 150 mg/m^2^/day on days 1–5, cytarabine 200 mg/m^2^/day on days 6–12, mitoxantrone 5 mg/m^2^/day on days 6–10, and intrathecal chemotherapy on day 6), induction 2 (HCEI: cytarabine 3 g/m^2^ every 12 hours on days 1–3, etoposide 100 mg/m^2^/day on days 1–5, idarubicin 10 mg/m^2^/day on day 1, and intrathecal chemotherapy on day 1), and 3 intensification therapy (HCM: cytarabine 2 g/m^2^ every 12 hours on days 1–5, mitoxantrone 5 mg/m^2^/day on days 1–3, and intrathecal chemotherapy on day 1; HCEI; and HCM) [7]. The chemotherapy promptly induced a first complete remission in the patient which has persisted 3.5 years from diagnosis without hematopoietic stem cell transplantation.

RNA sequencing on NextSeq500 (Illumina, Inc., CA, USA) was then used to screen for fusion partners, revealing a *KMT2A*-*MLLT10* rearrangement. The RNA analysis was conducted as follows: RNA was purified from the patient's bone marrow at diagnosis using ISOGEN (Nippon Gene Co., Ltd., Tokyo, Japan) according to the manufacturer's instructions. A sequencing library was then generated from 500 ng of total RNA using a TruSeq Stranded mRNA Library Prep Kit v2 (Illumina) according to the manufacturer's instructions. Next, sequencing was conducted at i-Laboratory LLP (Ibaraki, Japan). Obtained reads were aligned toward human genome assembly hg19, and fusion gene analysis was conducted with CLC Genomics Workbench Ver. 7.5.1 software (Qiagen, Venlo, Netherlands). Four sequencing tags supporting *KMT2A*-*MLLT10* rearrangement were obtained, which were then confirmed by Sanger sequencing. All sequencing data are shown in Table 1. Designed primers were 5′-TCAATTGCTGGCTCAGAAGA-3′ (*KMT2A* exon 5) and 5′-CTGAGCTATAAGAGCTGCCATT-3′ (*MLLT10* exon 16). The *KMT2A* breakpoint was located on intron 6, which was the upstream region of the sequencing primer for screening at diagnosis, and the *MLLT10* breakpoint was located on exon 14. Minimal residual disease (MRD) by reverse transcription-polymerase chain reaction (RT-PCR) was assessed from diagnosis as shown in Figure 1. Although MRD was positive at diagnosis, it changed to negative after induction therapy 1.

## 3. Discussion

Pediatric acute myeloid leukemia is classified by chromosomal and/or genetic abnormalities according to the World Health Organization Classification published in 2008. Chimeric genes including *KMT2A* rearrangements are used to predict disease outcome. *KMT2A* rearrangements are seen in about 20% of pediatric AML and are associated with poor outcome, while the disease outcome depends on its partner gene [1]. *KMT2A* has many partner genes, and each chimeric gene has a different prognosis.

It is difficult to quantify the effect of the partial duplication/amplification of *KMT2A* as the two patients previously reported had the same rearrangement of *KMT2A*-*MLLT10* with different treatment outcomes. A single abnormality within amplification of *KMT2A* is reported to have a gain-of-function effect for leukemogenesis [3]; however, the exact role of this “partial” duplication of *KMT2A* in acute leukemia is still unclear.

It is interesting to note that both previously reported and the current cases have the same rearrangement along with this partial duplication/amplification and diagnosis of FAB M5. Hence, we hypothesize that the leukemic cells that partially duplicate *KMT2A* tend to undergo *KMT2A*-*MLLT10* fusion and may act more similarly to *KMT2A*-*MLLT10* rearrangements caused by the insertion of *KMT2A* in chromosome 10p or an unbalanced translocation. This points to different causes producing the same prognostic effect. Similar rearrangement and the duplication of the 5′ segment of *KMT2A* might result in cell culture difficulties in other cases. Therefore, we can recommend using FISH assays to detect partial *KMT2A* duplication, and RNA sequencing may be useful to specify the fusion partner in such cases.

## 4. Conclusion

Partial duplication of the 5′ segment of *KMT2A* can be easily detected by FISH, but the crucial details of the *KMT2A*-*MLLT10* rearrangement may remain hidden from standard PCR testing, which might result in poor prognosis.

## Figures and Tables

**Table 1:** All sequencing data for breakpoint.

catcaaaccaattaaacctgtcactagaaacaaggcaccccaggaacctccagtaaagaaaggacgtcgatcgaggcggtgtgggcagtgtcccggctgccaggtgcctgaggactgtggtgtttgtacta
attgcttagataagcccaagtttggtggtcgcaatataaagaagcagtgctgcaagatgagaaaatgtcagaatctacaatggatgccttccaaagcctacctgcagaagcaagctaaag(*breakpoint*)
aggtatttataacagcaatgatgtagcagtatcgtttccaaatgtagtatctggctcgggatctagtactcctgtctccagctctcacttacctcagcagtcttctgggcatttgcaacaagtaggagcgctctctc
cctcagctgtgtcatctgcagcccctgctgttgctacaactcaggcaaatactctatctggatcttctctcagtcaggcaccatctcatatgtatggcaatagatcaaattcatcaatggcagctcttatagctcag
tctgaaaacaatcaaacag

Underlined sequencing was from *KMT2A*, and nonunderlined sequencing was from *MLLT10*.
